# Exploring the cardiometabolic profile of Emirati adults at risk for obstructive sleep apnea: findings from the UAE healthy future study

**DOI:** 10.3389/frsle.2026.1786010

**Published:** 2026-08-18

**Authors:** Manal Taimah, Amar Ahmad, Youssef Idaghdour, Raghib Ali, Abdishakur Abdulle, Mohammed Al-Houqani

**Affiliations:** 1 Public Health Research Center, New York University Abu Dhabi, Abu Dhabi, United Arab Emirates; 2 MRC Epidemiology Unit, University of Cambridge, Cambridge, United Kingdom; 3 Department of Medicine, College of Medicine and Health Sciences, UAE University, Al-Ain, United Arab Emirates

**Keywords:** cardiometabolic, inflammation, inflammatory marker, obstructive sleep apnea, renal biomarkers, young Emirati adults

## Abstract

**Background:**

Obstructive sleep apnea (OSA) is a significant contributor to cardiometabolic diseases, yet its burden and metabolic consequences remain underexplored in the United Arab Emirates (UAE). Additionally, little is known about how OSA risk relates to early metabolic and renal abnormalities in young adults. This study evaluated the association between OSA risk and a comprehensive profile of cardiometabolic and renal biomarkers in a large cohort of young Emirati adults.

**Methods:**

This cross-sectional study included 5,242 participants from the UAE Healthy Future Study. OSA risk was assessed using the STOP-Bang questionnaire and classified as no-to-low (score 0–2) or intermediate-to-high (score ≥3). Biomarkers assessed included HbA1c, lipid profile, ApoA, ApoB, C-reactive protein (CRP), serum creatinine, and urine microalbumin. Group differences were examined using *t*-tests and χ^2^ tests. Multivariable linear regression models adjusted for sociodemographic, lifestyle, and clinical covariates were used to assess associations between OSA risk and biomarker levels. Sensitivity analyses were conducted excluding participants with diabetes or dyslipidaemia.

**Results:**

The mean age was 27.5 years, and 30% of participants were classified as intermediate-to-high OSA risk. The intermediate-to-high OSA risk group exhibited greater adiposity, higher blood pressure, and more adverse cardiometabolic profiles than the no-to-low risk group. After adjustment, intermediate-to-high OSA risk was associated with elevated HbA1c (β = 0.18%, 95% CI: 0.14–0.23), higher total cholesterol, LDL-C, triglycerides, and ApoB, and lower HDL-C and ApoA (all *p* < 0.001). Inflammatory and renal markers were similarly elevated, including CRP (β = 0.14 mg/dL, 95% CI: 0.10, 0.18), serum creatinine (β = 0.11 mg/dL, 95% CI: 0.09, 0.13), and urine microalbumin (β = 3.61 mg/dL, 95% CI: 1.64, 5.60). Sex-stratified analyses showed higher median blood pressure and creatinine in males, and higher HDL-C and CRP in females. Sensitivity analyses produced consistent results for most biomarkers, except for urine microalbumin, which became non-significant.

**Conclusions:**

Elevated OSA risk was consistently associated with adverse cardiometabolic and renal profiles in young adults. These findings suggest that STOP-Bang may serve as a useful screening tool for early risk detection, warranting validation in longitudinal studies.

## Background

Obstructive sleep apnea (OSA) is a highly prevalent condition, strongly associated with increased risks for cardiovascular and metabolic disorders (Adderley et al., 2020; Gottlieb et al., 2010; Marin et al., 2005; Song et al., 2019). The global prevalence of OSA has risen dramatically alongside the obesity epidemic, affecting an estimated 936 million adults globally, with over 24 million cases identified in the United States alone (Lyons et al., 2020). The prevalence of OSA varies significantly due to factors such as gender, age and ethnicity (Fietze et al., 2019; Kaufmann et al., 2023; Lyons et al., 2020). In the United Arab Emirates (UAE), the estimated prevalence of high OSA risk is 16.58%, with a significantly higher burden among men (26.46%) compared to women (4.10%) among Emirati adults (Taimah et al., 2024).

OSA is characterized by recurrent episodes of partial or complete airway obstruction, leading to hypopnea (shallow breathing) and apnea (cessation of breathing; Rundo, 2019). These events result in intermittent oxygen desaturation, hypercapnia, and frequent micro-arousals (Zhao et al., 2019). OSA is significantly linked to major cardiometabolic diseases such as hypertension, dyslipidaemia, and hyperglycaemia (Adderley et al., 2020; Gottlieb et al., 2010; Marin et al., 2005). The physiological mechanisms linking OSA to cardiometabolic dysfunction are well-documented, including intermittent hypoxia, sympathetic activation, and systemic inflammation (Chopra et al., 2017; Lavie, 2012; Song et al., 2019; Tamisier et al., 2011). Existing research has investigated the relationships between individual cardiometabolic disorders (hypertension, diabetes, or dyslipidaemia). However, the concurrent association between OSA and multiple cardiometabolic abnormalities remains incompletely understood, particularly within specific populations.

Numerous Western studies have demonstrated that OSA severity is positively correlated with the degree of cardiometabolic impairment (Geovanini et al., 2018; Wu et al., 2022; Zhao et al., 2019). Specifically, the apnea-hypopnea index (AHI), a key measure of OSA severity, has been shown to be significantly associated with markers of metabolic syndrome, including elevated blood pressure, fasting glucose, triglycerides, low-density lipoprotein cholesterol (LDL-C), Apolipoprotein B (ApoB) and reduced high-density lipoprotein cholesterol (HDL-C) and lower Apolipoprotein A (ApoA) cholesterol levels (Geovanini et al., 2018; Zhao et al., 2019). Several risk factors, including obesity, craniofacial anatomical structure, physical inactivity, and smoking, further contribute to this association, highlighting its multifactorial nature (Hall et al., 2020; Lee et al., 2010; Lui et al., 2016; Zdravkovic et al., 2022).

The UAE faces significant public health challenges due to rapid urbanization and rising rates of non-communicable diseases (Elmusharaf et al., 2021). The high obesity rate in Emirati adults, estimated at 37%, is an important factor that may increase the risk of OSA and metabolic dysfunction [The Ministry of Health and Prevention (MoHAP), 2023]. Cardiovascular disease, the leading cause of mortality in the UAE, may be exacerbated by underdiagnosed OSA-related cardiometabolic risks (World Health Organization, 2023). Moreover, the unique cultural and environmental factors, such as diets rich in calorie-dense foods, sedentary lifestyles, and potential genetic susceptibilities, probably influence the cardiometabolic risk of Emirati adults (World Health Organization, 2023).

While global evidence demonstrates associations between OSA and multiple cardiometabolic abnormalities, data from Middle Eastern settings remain limited. No prior research has examined how OSA risk corresponds to detailed cardiometabolic and renal markers in young Emirati adults—a population undergoing significant lifestyle and epidemiological transitions. This study addresses this gap by providing an initial, detailed description of health profiles across OSA risk categories, offering context-specific insights that may support early risk assessment and guide future analytical or longitudinal investigations in this population.

## Material and methods

### Study Design and participants

This cross-sectional analysis utilized baseline data from the UAE Healthy Future Study (UAEHFS), gathered between February 2016 and March 2023. The UAEHFS is an ongoing longitudinal study aiming to recruit 20,000 Emirati nationals to enhance understanding of the causes and risk factors for non-communicable diseases (Abdulle et al., 2018). The study was conducted across several recruitment centers in Abu Dhabi, Al-Ain, Dubai, Sharjah, and Ras Al Khaimah. The UAEHFS enrolled eligible Emirati adults aged 18 years and above who were able to provide consent. Pregnant women and non-Emirati individuals were excluded. Convenience sampling was employed, inviting participants to the study recruitment centers across the UAE. All participants reviewed a detailed information leaflet and were allowed to ask questions before providing their written informed consent. On arrival, participants completed an online survey (available in Arabic or English), had their physical and anthropometric measurements taken, and provided biological samples. Detailed methodology for the UAEHFS is available in previously published studies (Abdulle et al., 2018; UAEHFS, 2025). An anonymised electronic dataset, devoid of personal identifiers, was used for the present analysis to address the research question. The current analysis included 5,242 (37%) participants out of 14,308, who completed the STOP-Bang questionnaire and provided body measurements and blood samples.

### Ethical considerations

The UAEHFS was conducted in accordance with the Declaration of Helsinki and the Abu Dhabi Department of Health standards for research involving human subjects (Abdulle et al., 2018; UAEHFS, 2025). Several Institutional Review Boards, including the Abu Dhabi Health Research and Technology Committee (DOH/HQD/2020/516), the New York University Abu Dhabi Institutional Review Board, the Dubai Scientific Research Ethics Committee, and the UAE Ministry of Health and Prevention Higher Committee of Ethics, approved the study protocols. All participants read and understood the information leaflet and signed the consent form before recruitment.

### Exposure parameter

OSA risk was assessed using the STOP-Bang questionnaire, a validated tool comprising eight items: snoring, tiredness, observed apnea, high blood pressure, body mass index (BMI), age, neck circumference, and gender (Chung et al., 2008). Healthcare professionals use this tool to identify individuals at risk for OSA (Jang et al., 2023). The STOP-Bang questionnaire shows high sensitivity in identifying low, intermediate, and high OSA risk (89.1%, 90.7%, and 93.9%), respectively (Hwang et al., 2021). The Arabic version of the STOP-Bang questionnaire was assessed and validated (Alhouqani et al., 2015). In this study, participants were classified into two OSA risk categories based on STOP-Bang questionnaire scores: 0–2 indicated no-to-low risk, and 3 or higher indicated intermediate-to-high risk.

### Definition and data collection of cardiometabolic outcomes

Systolic blood pressure (SBP) and diastolic blood pressure (DBP) were measured in a sitting position twice after having a rest for at least 5 min using the SphygmoCor XCEL System blood pressure monitor (two measurements separated by 2 min at rest). The average of the two readings was used for this analysis. Blood pressure was used as a continuous variable in the regression analysis. Central mean pressure (CMP), which reflects average pressure in a cardiac pulse, was directly measured by the SphygmoCor XCEL System. The average of the two readings was used for this analysis. Plasma or serum samples were used to assess cardiometabolic parameters, including glycated hemoglobin A1C (HbA1c), fasting/random glucose, total cholesterol, HDL-C, LDL-C, and triglycerides, ApoA, ApoB, and C-reactive Protein. All measurements were performed on the DxC600 analyser. All results were calibrated against traceable standards for quality control, in accordance with standard procedures (Abdulle et al., 2018). HbA1c was measured using an immunoturbidimetric assay, which quantifies glycated hemoglobin as a percentage of total hemoglobin. Glucose was measured *via* the hexokinase method. Total cholesterol, HDL-C, LDL-C, and triglycerides were analyzed enzymatically; HDL-C and LDL-C were directly measured (Abdulle et al., 2018). C-Reactive protein (CRP) levels were determined using an immunoturbidimetric assay where antigen-antibody complexes form aggregates proportional to CRP concentration.

### Anthropometric measurements

Neck circumference, with cut-off values of 43 cm for males and 41 cm for females, was used to calculate the STOP-Bang score (Abdulle et al., 2018; Chung et al., 2008). Body composition and BMI were calculated using the Tanita MC-780 MA body composition analyser, and the cut-off value of 30 kg/m^2^ was used to calculate the STOP-Bang score. Waist-to-hip ratio was calculated by dividing waist circumference by hip circumference, both measured using a non-stretchable Seca-200 tape.

### Demographic and lifestyle parameters

Participants' sociodemographic characteristics, including age, sex, marital status, education, employment status, and family history, were assessed. Age and sex were self-reported and verified using the Emirate ID card. Marital status was categorized as Single, Married, or Other. Educational attainment was categorized into four groups based on self-reports of the highest level of education completed: middle school or less includes individuals who did not complete secondary education, corresponding to Grade 9 or below; secondary school includes those who completed high school (Grades 10–12) and obtained a General Secondary Education Certificate but did not pursue higher education; university or more includes individuals who completed higher education of university or higher. Employment status was categorized into three groups based on participants' self-reported employment as not employed, employed, or a student. Family history was assessed by asking participants if any of their parents suffer from cardiovascular diseases or type 2 diabetes mellitus (T2DM). Participants' medical history was assessed by asking the participants if they had a history of T2DM, hypertension and dyslipidaemia. Participants indicated their sleep duration (hours slept in a 24-h period), which was categorized according to the American Academy of Sleep Medicine guidelines as short sleep (< 7 h) or recommended sleep (≥7 h; Watson, 2015). Smoking was assessed by asking participants about their current smoking status and whether they had smoked more than 100 cigarettes in their entire lives. Participants were classified as smokers if they had smoked over 100 cigarettes in their lifetime and if they currently smoke any of the following at least once a month: cigarettes, midwakh/dokha, or shisha/waterpipe.

### Statistical analysis

Baseline characteristics were described using means and standard deviations for continuous variables, and frequencies with percentages for categorical variables. Differences between OSA risk groups (no-to-low vs. intermediate-to-high) were assessed using independent two-sample *t*-tests for continuous variables and chi-square tests for categorical variables. Univariate linear regression models were used to examine the relationships between OSA risk groups and individuals' cardiometabolic factors, including HbA1c, total cholesterol, LDL-C, HDL-C, triglycerides, ApoA, ApoB, serum creatinine, CRP and urine microalbumin. Each cardiometabolic variable was treated as an outcome, with the OSA risk group as the predictor. Multivariable linear regression models were then fitted to assess adjusted associations between OSA risk and each cardiometabolic outcome. Models included marital status, education, employment, smoking status, and sleep duration as covariates. HbA1c models were additionally adjusted for history of diabetes or hypoglycaemic medication use, and lipid variables were adjusted for use of lipid-lowering medication (Edwards et al., 2020; Etindele Sosso and Matos, 2021; Zhao et al., 2019). A sensitivity analysis was conducted by excluding participants with a history of T2DM or dyslipidaemia. As this study was a secondary analysis of the UAE Healthy Future Study, no *a priori* sample size calculation was performed specifically for the present analyses. However, the analytical sample included 5,242 participants, which substantially exceeded the minimum sample size recommendations for multivariable regression modeling relative to the number of predictor parameters included, supporting stable parameter estimation and adequate statistical power to detect meaningful associations (Harrell, 2015). Stata version 18 was used for all statistical analyses (StataCorp, 2023).

## Results

Among the total cohort of 14,308 participants, 5,242 adult Emirati participants were included in the analysis, with 69.9% (*n* = 3,668) classified as having no-to-low risk for OSA, and 30.03% (*n* = 1,574) categorized as having intermediate-to-high risk for OSA. The remaining participants were excluded due to incomplete STOP-Bang data or missing biomarker measurements.

### Sociodemographic characteristics

Table 1 presents the sociodemographic characteristics of the study participants, presented as means with standard deviations. Categorical variables were summarized using frequencies and percentages. Participants in the intermediate-to-high OSA risk group were older than those in the no-to-low risk group (mean age 31.2 vs. 26.0 years, *p* < 0.001). Males comprised a significantly greater proportion of the intermediate-to-high risk group (87.8%) than of the no-to-low risk group (47.8%). Marital status also differed markedly. A higher proportion of the intermediate-to-high risk group was married (52.4% vs. 29.1%), while the no-to-low risk group had a higher proportion of single individuals (68.4% vs. 44.1%). Educational attainment and employment status also varied by OSA risk level. Participants in the intermediate-to-high risk group were more likely to have completed only middle school or less (7.4% vs. 2.7%) and to be employed (73.7% vs. 52.1%). In contrast, those in the no-to-low risk group included a higher proportion of students (30.1% vs. 10.2%; *p* < 0.001 for both comparisons).

**Table 1:** Baseline characteristics of Emirati adults based on obstructive sleep apnea (OSA) risk.

Characteristic	No-to-low OSA risk group	Intermediate-to-high OSA risk group	Total population	*P* value
*N*	3,668 (69.87%)	1,574 (30.03%)	5,242	
Demographics				
*Age* (years), mean (SD)	26.0 (7.5)	31.2 (9.5)	27.5 (8.5)	< 0.001
Sex, n (%)				< 0.001
Male	1,753 (47.8)	1,382 (87.8)	3,135 (59.8)	
Female	1,915 (52.2)	192 (12.2)	2,107 (40.2)	
Marital status, n (%)				< 0.001
Single	2,492 (68.4)	688 (44.1)	3,180 (61.1)	
Married	1,058 (29.1)	817 (52.4)	1,875 (36.1)	
Others	91 (2.5)	55 (3.5)	146 (2.8)	
Unknown	27	14	41	
Educational attainment, n (%)				< 0.001
Middle school or less	95 (2.7)	113 (7.4)	208 (4.1)	
Secondary school	1,590 (45.4)	645 (42.5)	2,235 (44.5)	
University or more	1,819 (51.9)	761 (50.0)	2,580 (51.4)	
Unknown	164	55	219	
Employment, n (%)				< 0.001
Not employed	539 (17.7)	218 (16.1)	757 (17.3)	
Employed	1,581 (52.1)	996 (73.7)	2,577 (58.8)	
Student	914 (30.1)	138 (10.2)	1,052 (24.0)	
Unknown	634	222	856	
Family history of CVD & T2DM, n (%)				< 0.001
No	1,445 (39.6)	450 (28.8)	1,895 (36.4)	
Yes	2,204 (60.4)	1,113 (71.2)	3,317 (63.6)	
Unknown	19	11	30	
Medical History (Yes), n (%)				
Hypertension	121 (3.6)	352 (24.2)	473 (9.7)	< 0.001
Type 2 diabetes mellitus	102 (3.0)	128 (8.7)	230 (4.7)	< 0.001
Dyslipidaemias	217 (12.2)	194 (28.5)	411 (17.1)	< 0.001
Lifestyle factors
Smoking status, n (%)				< 0.001
Non-smoker	2,825 (77.4)	844 (53.9)	3,669 (70.4)	
Smoker	823 (22.6)	723 (49.4)	1,546 (31.5)	
Sleeping hours, n (%)				< 0.001
< 7 h	1,205 (34.2)	603 (40.7)	1,808 (36.2)	
>= 7 h	2,314 (65.8)	879 (59.3)	3,193 (63.9)	
Unknown	149	92	241	

Values are presented as mean (standard deviation) for continuous variables and number (percentage) for categorical variables. P-values correspond to comparisons between the no-to-low and intermediate-to-high OSA risk groups using t-tests for continuous variables and χ^2^ tests for categorical variables. OSA risk was assessed using the STOP-Bang questionnaire and categorized as no-to-low risk (score 0–2) or intermediate-to-high risk (score ≥3).

OSA, obstructive sleep apnea; CVD, cardiovascular diseases; T2DM, type 2 diabetes mellitus.

Unknown includes answers of “I don't know”, “Prefer not to answer” and missing data.

Percentages do not include the Unknown category.

### Medical history and lifestyle factors

Individuals in the intermediate-to-high OSA risk group were more likely to have T2DM than those in the no-to-low risk group (8.7% vs. 3.0%; see Table 1). A family history of cardiovascular diseases or T2DM was also more common among those at higher OSA risk (71.2% vs. 60.4%, *p* < 0.001). Lifestyle differences were evident, with 49.4% of those at higher risk being smokers compared to 22.6% in the no-to-low risk group (*p* < 0.001). Additionally, short sleep duration (< 7 h per day) was more prevalent among the intermediate-to-high risk group (40.7% vs. 34.2%, *p* < 0.001).

### Cardiometabolic and anthropometric profiles

Table 2 presents the anthropometric and cardiometabolic characteristics of participants by OSA risk group. Individuals in the intermediate-to-high OSA risk group had significantly higher measures of adiposity, including larger neck circumference (39.6 vs. 34.5 cm), higher waist-to-hip ratio (0.89 vs. 0.81), and higher BMI (31.3 kg/m^2^ vs. 25.1 kg/m^2^; all *p* < 0.001). Cardiometabolic parameters were also notably elevated in the intermediate-to-high OSA risk group compared to the no-to-low risk group. Mean systolic and diastolic blood pressure were markedly elevated (136.4 vs. 121.5 mmHg and 84.8 vs. 75.6 mmHg, respectively), as was central mean pressure (101.6 vs. 91.0 mmHg; all *p* < 0.001). Glycaemic markers were higher in the intermediate-to-high risk group, including HbA1c (5.5 vs. 5.2%), fasting glucose (97.3 vs. 90.9 mg/dL), and random glucose (104.2 vs. 92.6 mg/dL; all *p* < 0.001). Lipid profiles also differed significantly: total cholesterol, LDL-C, fasting and non-fasting triglycerides, and ApoB were higher in the intermediate-to-high OSA risk group, whereas HDL-C and ApoA were lower (all *p* < 0.001). Supplementary Figure S1 further illustrates the distribution of dyslipidaemia by OSA status, displaying both counts and percentages across risk groups. Inflammatory and renal markers showed the same pattern, with higher CRP (0.44 vs. 0.30 mg/dL), serum creatinine (0.84 vs. 0.70 mg/dL), and urine microalbumin (5.7 vs. 1.6 mg/dL; all *p* < 0.001) among those at higher OSA risk.

**Table 2:** Cardiometabolic and anthropometric parameters by obstructive sleep apnea (OSA) risk group.

Characteristic, mean (SD)	No-to-low OSA risk group, (*n* = 3,668)	Intermediate-to-high OSA risk group, (*n* = 1,574)	Total population (*n* = 5,242)	*P* value
Anthropometric measurements				
Neck circumference	34.5 (3.7)	39.6 (3.4)	35.9 (4.3)	< 0.001
Waist-to-hip ratio	0.81 (0.08)	0.89 (0.07)	0.84 (0.09)	< 0.001
BMI	25.1 (5.2)	31.3 (7.0)	26.9 (6.4)	< 0.001
Cardiometabolic parameters				
Systolic BP (mmHg)	121.5 (11.9)	136.4 (14.2)	125.9 (14.4)	< 0.001
Diastolic BP (mmHg)	75.6 (8.5)	84.8 (10.7)	78.3 (10.1)	< 0.001
Central mean pressure (mmHg)	91.0 (9.3)	101.6 (11.6)	94.2 (11.1)	< 0.001
HbA1c (%)	5.2 (0.5)	5.5 (0.9)	5.3 (0.7)	< 0.001
Fasting blood glucose (mg/dL)	90.9 (20.6)	97.3 (22.9)	92.9 (22.9)	< 0.001
Random blood sugar (mg/dL)	92.6 (26.2)	104.2 (47.1)	96.0 (34.3)	< 0.001
Total cholesterol (mg/dL)	181.4 (35.1)	194.6 (39.5)	185.3 (37.0)	< 0.001
LDL cholesterol (mg/dL)	110.9 (31.9)	125.8 (35.7)	115.4 (33.8)	< 0.001
HDL Cholesterol (mg/dL)	52.3 (13.0)	44.2 (11.2)	49.9 (13.1)	< 0.001
Triglycerides (fasting; mg/dL)	69.6 (43.7)	112.8 (92.8)	83.0 (66.0)	< 0.001
Triglycerides (non-fasting; mg/dL)	92.0 (66.4)	147.3 (107.3)	108.2 (84.5)	< 0.001
Apolipoprotein A (mg/dL)	141.5 (22.9)	133.6 (21.6)	139.2 (22.8)	< 0.001
Apolipoprotein B (mg/dL)	85.3 (23.2)	99.4 (26.7)	89.5 (25.2)	< 0.001
Serum creatinine (mg/dL)	0.70 (0.2)	0.84 (0.4)	0.74 (0.3)	< 0.001
Serum CRP (mg/dL)	0.30 (0.5)	0.44 (0.6)	0.33 (0.5)	< 0.001
Urine microalbumin (mg/dL)	1.6 (7.6)	5.7 (36.6)	2.9 (21.3)	< 0.001

Values are presented as mean (standard deviation). P-values correspond to comparisons between the no-to-low and intermediate-to-high OSA risk groups using t-tests. OSA risk was assessed using the STOP-Bang questionnaire and categorized as no-to-low risk (score 0–2) or intermediate-to-high risk (score ≥ 3).

BP, blood pressure; HbA1c, glycated hemoglobin A1C; LDL-C, low-density lipoprotein cholesterol; HDL-C, high-density lipoprotein cholesterol; CRP, C-reactive protein.

Fasting blood glucose and fasting triglyceride analyses included 1,105 participants.

Urine microalbumin was measured for 2,580 participants.

### Associations between OSA risk and cardiometabolic markers

Table 3 presents the linear regression analyses examining associations between OSA risk and cardiometabolic parameters. Among Emirati adults, intermediate-to-high OSA risk was consistently associated with adverse cardiometabolic profiles after controlling for key sociodemographic and clinical covariates.

**Table 3:** Associations between OSA risk and cardiometabolic outcomes in adjusted linear regression models.

Variables	Adjusted estimate model (95% CI)	*p*-value
Glycemic control		
HbA1c (%)	0.18 (0.14, 0.23)	< 0.001
Lipid profile		
Total Cholesterol (mg/dL)	7.18 (4.53, 9.84)	< 0.001
LDL-C (mg/dL)	9.44 (7.01, 11.86)	< 0.001
HDL-C (mg/dL)	−7.16 (−8.12, −6.22)	< 0.001
Triglycerides (mg/dL)	0.41 (0.32, 0.50)	< 0.001
Apolipoproteins		
ApoA (mg/dL)	−7.93 (−9.59, −6.28)	< 0.001
ApoB (mg/dL)	11.28 (9.54, 13.02)	< 0.001
Inflammation		
CRP (mg/dL)	0.14 (0.10, 0.18)	< 0.001
Kidney markers		
Serum creatinine (mg/dL)	0.11 (0.09, 0.13)	< 0.001
Urine microalbumin (mg/dL)	3.61 (1.64, 5.60)	< 0.001

Estimates represent mean differences (95% confidence intervals) in biomarker levels for individuals with intermediate-to-high OSA risk compared with those at no-to-low risk, based on STOP-Bang classification.

Adjusted estimate model: Adjusted for marital status, education, employment, smoking status, and sleep duration. HbA1c models were additionally adjusted for history of diabetes, and lipid variables were adjusted for use of lipid-lowering medication.

Triglyceride was log-transformed, and regression analysis was done for fasting participants (n = 776).

HbA1c, glycated hemoglobin A1C; LDL-C, low-density lipoprotein cholesterol; HDL-C, high-density lipoprotein cholesterol; CRP, C-reactive protein.

Participants with intermediate-to-high OSA risk exhibited consistently poorer cardiometabolic profiles than those at low risk. After adjustment, higher OSA risk was associated with elevated HbA1c (β = 0.18%, 95% CI: 0.14–0.23) and adverse lipid parameters (all *p* < 0.001). Furthermore, OSA risk showed significant positive associations with inflammatory and renal markers, including CRP (β = 0.14 mg/dL, 95% CI: 0.10, 0.18), serum creatinine (β = 0.11 mg/dL, 95% CI: 0.09, 0.13), and urine microalbumin (β = 3.61 mg/dL, 95% CI: 1.64, 5.60).

**Figure 1:**
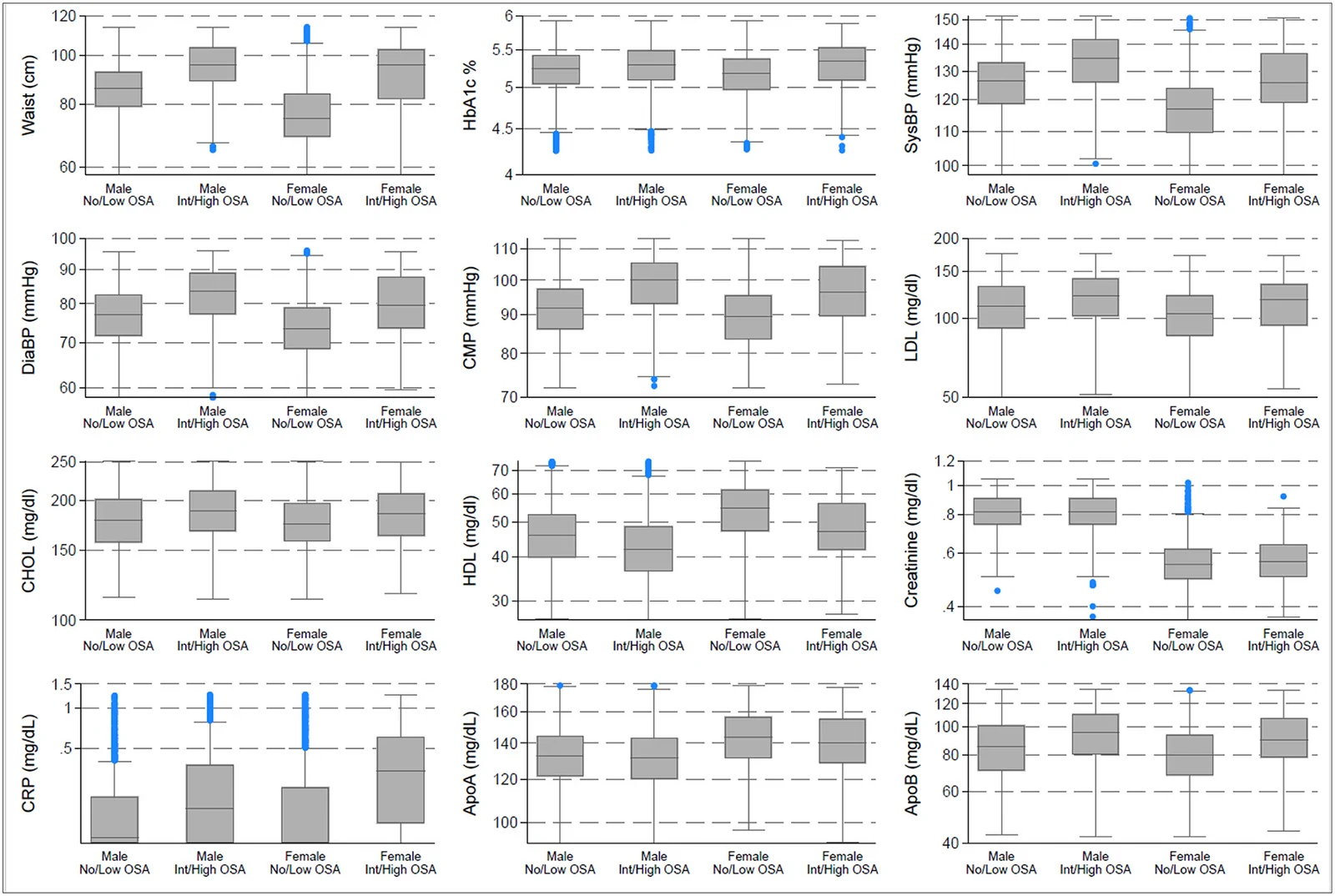
*Sex-specific differences in cardiometabolic parameters by obstructive sleep apnea (OSA) risk status*. Distributions of cardiometabolic biomarkers are presented separately for males and females across OSA risk groups. Notable patterns include elevated blood pressure and LDL-C levels among males with intermediate-to-high OSA risk, and consistently higher HDL-C levels among females across all risk categories. Abbreviations: LDL, low-density lipoprotein cholesterol; TC, total cholesterol; HDL, high-density lipoprotein cholesterol; HbA1c, hemoglobin A1c; SysBP, systolic blood pressure; DiaBP, diastolic blood pressure; CMP, central mean pressure; CRP, C-reactive protein; ApoA, apolipoprotein A; ApoB, apolipoprotein B. Clinical thresholds values: Waist circumference; male <94 cm, female <80 cm (World Health Organization, 2011); HbA1c <5.7% (normal), 5.7%−6.4% (prediabetes), ≥6.5% (diabetes; American Diabetes Association Professional Practice Committee, 2022); BP >120/80 mmHg (Magder, 2018); LDL<100, HDL; male>40/, female >50, CHOL<200 mg/dL; Creatinine male 0.74–1.35, female 0.59–1.04 mg/dL; CRP<1.0 mg/dL; ApoA 110–180, ApoB<90 mg/dL (Grundy, 2019).

Figure 1 shows Sex-stratified box plots of key biomarkers, highlighting greater SBP, DBP, and creatinine in males, and higher HDL-C and CRP in females.

### Sensitivity analysis

Sensitivity analyses excluding participants with T2DM or dyslipidaemia (*n* = 1,982) yielded results consistent with the primary findings (Supplementary Table S1). Intermediate-to-high OSA risk remained significantly associated with higher HbA1c, total cholesterol, LDL-C, fasting triglycerides, and ApoB, as well as lower HDL-C and ApoA (all *p* < 0.05). Elevated inflammatory and renal markers also persisted, with significant increases in CRP and serum creatinine among the intermediate-to-high risk group. Associations with urine microalbumin were not statistically significant in the restricted sample. Overall, these findings suggest that the observed cardiometabolic and renal differences are not driven solely by individuals with established metabolic disease and remain evident even in a metabolically healthier subset.

## Discussion

This study highlights the association between OSA risk and adverse cardiometabolic profiles among young adults in the UAE, a population facing a growing burden of non-communicable diseases yet underrepresented in sleep research. Our principal finding is that individuals at intermediate-to-high OSA risk showed significantly poorer cardiometabolic health across several parameters, including higher adiposity and blood pressure, impaired glycaemic control, atherogenic dyslipidaemia, systemic inflammation, and early renal dysfunction compared to the no-to-low OSA risk group. In our cohort, 30.03% of participants were classified as intermediate-to-high risk, with a notable sex difference. These observations suggest that OSA may be an under-recognized factor contributing to the early emergence of cardiometabolic disturbances within this young population.

Males accounted for 87.8% of the intermediate-to-high risk group, a distribution that aligns with widely reported epidemiological patterns indicating higher OSA risk among men (Fietze et al., 2019; Martins and Conde, 2021; Messineo et al., 2024). The strong male predominance in our intermediate-to-high risk group aligns with established literature and is likely attributable to anatomical factors like greater central obesity and neck circumference, as well as relative progesterone deficiency compared to premenopausal women (Bonsignore et al., 2013; Martins and Conde, 2021). Within this at-risk demographic, we documented significant cardiometabolic disturbances. Specifically, we found elevated HbA1c levels (Tables 2 and 3), building on previous evidence of a significant association between OSA and HbA1c independent of age, sex, and BMI (López-Cepero et al., 2024). This supports the pathophysiological evidence that OSA-related intermittent hypoxia and sleep fragmentation can directly impair glycemic control and insulin sensitivity (Huang et al., 2022; Lavie, 2012).

Our analysis further revealed a clinically significant dyslipidaemic profile characterized by elevations in LDL-C (14.9 mg/dL), total cholesterol (13.2 mg/dL), and triglycerides (43.2 mg/dL), alongside a reduction in protective HDL-C (8.1 mg/dL) in the intermediate-to-high OSA risk group (all *p* < 0.001; Table 2). These findings are consistent with previous evidence from the Sleep Heart Health Study, which linked OSA severity to atherogenic dyslipidaemia (Newman et al., 2001; Zhao et al., 2019). However, in contrast to the Multi-Ethnic Study of Atherosclerosis, the associations between total cholesterol and triglycerides became nonsignificant after adjustment for demographic and clinical confounders (Geovanini et al., 2018). This discrepancy may arise from our population's younger age, which may detect metabolic disturbances earlier before compensatory mechanisms come into play, as indicated by the significant unadjusted effects in the Multi-Ethnic Study of Atherosclerosis.

This study found that systemic inflammation, marked by elevated CRP and changes in apolipoproteins, further defines the high-risk profile. The statistically significant decrease in ApoA and increase in ApoB, which remained significant after adjustment, provide a more nuanced picture of possible atherogenic risk than standard lipids alone. The proposed mechanism, in which intermittent hypoxia can suppress ApoA and promote ApoB expression in hepatocytes, provides a direct link between OSA and accelerated atherogenesis (Bikov et al., 2021; Cao et al., 2020; Fadaei et al., 2022). Equally concerning are the signs of early renal impairment, marked by elevated serum creatinine and a nearly four-folds increase in urine microalbumin. This likely results from the dual insults of OSA-induced oxidative stress, renal vasoconstriction, and hypertension-mediated glomerular hyperfiltration (Fu et al., 2016; Pack and Gislason, 2009). These findings suggest that OSA risk may be associated with early organ changes; however, the causal direction cannot be determined from this cross-sectional study design. Importantly, although many mean biomarker values remained within clinically normal ranges, the consistent differences observed between OSA risk groups may reflect early subclinical cardiometabolic alterations rather than established disease. Given the relatively young age of the cohort, these findings may represent an earlier stage in the progression of cardiometabolic dysfunction (Chou et al., 2011; Zamarrón et al., 2021). It is also important to note that several components of the STOP-Bang questionnaire, including age, sex, BMI, neck circumference, and hypertension, are established cardiometabolic risk factors. Therefore, some of the observed associations may reflect the broader cardiometabolic risk profile captured by the screening tool rather than the independent effects of sleep-disordered breathing. Nevertheless, the objective of this study was to characterize cardiometabolic profiles across OSA risk groups identified using a validated screening instrument, rather than to isolate OSA's independent contribution.

Significant demographic and behavioral disparities contextualize the observed cardiometabolic burden. Participants with intermediate-to-high OSA risk were significantly more likely to report short sleep duration, current smoking, and lower educational attainment. This clustering of risk factors suggests a syndemic relationship, where social disadvantage (e.g., lower education) and unhealthy behaviors (e.g., smoking, short sleep) interact to amplify OSA vulnerability and its metabolic consequences (Bakker et al., 2015; Etindele Sosso and Matos, 2021; Jang et al., 2023; Lui et al., 2016). The higher proportion of married participants with increased OSA risk may be linked to partner-related signs recognition, such as snoring and airway obstruction. Conversely, the higher proportion of single individuals in the no-to-low risk group may have reduced opportunities to recognize and report symptoms, potentially influencing questionnaire-based risk classification. Furthermore, the significant association with self-reported family history of cardiometabolic disease, while potentially underestimated, warrants investigation into gene-environment interactions in Arab populations, where consanguinity may modify hereditary risk.

## Strengths and limitations

This study should be interpreted in the context of its strengths and limitations. A key strength is the comprehensive assessment of cardiometabolic risk, achieved by evaluating a broad panel of biomarkers, including lipids, glycaemic markers, serum creatinine, microalbumin, and blood pressure, which collectively provide a multifaceted view of the association between OSA risk and early cardiometabolic and renal dysfunction. The lipid assessment protocol, utilizing both fasting and non-fasting samples aligned with contemporary guidelines, enhanced participant convenience and study feasibility without compromising clinical relevance for most participants, while reserving confirmatory fasting tests for indicated cases (Nordestgaard et al., 2016). Second, this investigation addresses a critical evidence gap by focusing on a young adult population (mean age 27.5 years), offering valuable insights into the early, subclinical stages of OSA-related metabolic impairment in a demographic that is often underrepresented in sleep research. The sex-inclusive design improves the generalisability of findings beyond male-only cohorts. As one of the first studies to explore these associations in this specific demographic context, the findings can hold direct public health relevance for the UAE and may offer insights for neighboring regions with similar sociodemographic and clinical profiles.

Several limitations warrant consideration. First, the cross-sectional design precludes causal inferences, and the use of convenience sampling may limit the sample's representativeness of the broader Emirati population, despite its size and geographic diversity. OSA risk was assessed using the validated STOP-Bang questionnaire, a pragmatic tool for large-scale studies that integrates both objective measures and subjective symptoms. However, the absence of polysomnography, the diagnostic gold standard, may lead to misclassification of OSA risk status. While STOP-Bang has high sensitivity for identifying individuals at elevated risk of OSA, its lower specificity may lead to misclassification, potentially overestimating OSA risk in population-based settings. Information on antihypertensive medication use was not incorporated into the present analysis. Therefore, the potential impact of antihypertensive treatment on the observed cardiometabolic profiles may not have been fully captured. Additionally, low variation explained by the OSA risk in all cardiometabolic outcomes (approximately 10%) suggests that OSA risk is one of several factors influencing cardiometabolic health, with unmeasured confounders such as diet and physical activity likely contributing. Because OSA risk was modeled as a binary variable, the regression coefficients from both univariate and multivariable linear models represent mean differences in biomarker levels between the intermediate-to-high and no-to-low risk groups. This clarification is important for accurately interpreting the magnitude of the reported associations. Consequently, the generalisability of these findings to older populations or other ethnic groups may be limited. Despite these limitations, this study provides important preliminary evidence underscoring the potential value of early screening for cardiometabolic dysfunction in young adults at risk for OSA. Unmeasured lifestyle and dietary factors, as well as genetic predispositions, may have influenced cardiometabolic profiles and should be considered in future studies.

## Conclusions

This study identifies significant cross-sectional associations between elevated OSA risk, as measured by the STOP-Bang questionnaire, and adverse cardiometabolic and renal profiles in young Emirati adults. Individuals at intermediate-to-high OSA risk exhibited a consistent pattern of subclinical multisystem alterations, including higher blood pressure, atherogenic lipid patterns, poorer glycaemic control, elevated inflammatory markers, and early indications of renal dysfunction.

Although the cross-sectional design limits causal interpretation, the breadth and consistency of these associations suggest that OSA risk affecting more than 30% of this cohort may serve as a practical indicator of concurrent metabolic and vascular vulnerability. In a population experiencing rising rates of non-communicable diseases, incorporating brief OSA screening tools, such as STOP-Bang, into routine preventive assessments may help identify individuals who could benefit from further cardiometabolic evaluation or lifestyle modification. However, such approaches should be implemented cautiously and supported by additional evidence.

Future longitudinal studies employing objective diagnostic measures, such as polysomnography, are essential to clarify temporal relationships, strengthen causal understanding, and determine whether early detection and management of OSA can modify the progression toward overt cardiometabolic or renal disease. Overall, these findings position OSA risk as a potential early marker of broader systemic health in young adults, warranting continued investigation in this understudied population.

## Data Availability

The datasets analysed during this study are not publicly available due to UAEHFS data governance policies but may be available from the UAEHFS upon reasonable request and approval.
