# Supplementary material for: Exploring the cardiometabolic profile of Emirati adults at risk for obstructive sleep apnea: findings from the UAE healthy future study
Source: Front Sleep. 2026 Aug 18;5:1786010. doi: 10.3389/frsle.2026.1786010 (PMC13529505; doi:10.3389/frsle.2026.1786010)
Supplement: Supplementary file 1 [file Data_Sheet_1.docx]

**Exploring the Cardiometabolic Profile of Emirati Adults at Risk for Obstructive Sleep Apnea: Findings from the UAE Healthy Future Study**

Manal Taimah^1^, Amar Ahmad^1^, Youssef Idaghdour^1^, Raghib Ali^1&2^, Abdishakur Abdulle^1^, Mohammed Al-Houqani^3*^

^1^ Public Health Research Center, New York University Abu Dhabi, Abu Dhabi, United Arab Emirates

^2^ MRC Epidemiology Unit, University of Cambridge, Cambridge CB2 1TN, UK

^3^ Department of Medicine, College of Medicine and Health Sciences, UAE University, Al-Ain, United Arab Emirates

**Supplementary Table S1. Sensitivity analysis: Associations between OSA Risk and Cardiometabolic Outcomes in Unadjusted and Adjusted Linear Regression Models after excluding participants with type 2 diabetes or dyslipidaemia (n=1982)**

| **Outcome** | **Model 1: Unadjusted Estimate (95% CI)** | **p-value** | **Model 2: Adjusted Estimate (95% CI)** | **p-value** |
| --- | --- | --- | --- | --- |
| **Glycaemic Marker** |  |  |  |  |
| HbA1c (%) | 0.094 (0.054, 0.133) | <0.001 | 0.086 (0.035, 0.136) | 0.001 |
| **Lipid Profile** |  |  |  |  |
| Total Cholesterol (mg/dL) | 2.54 (0.22, 4.87) | 0.032 | 3.77 (0.92, 6.62) | 0.010 |
| LDL-C (mg/dL) | 5.55 (3.28, 7.82) | <0.001 | 6.23 (3.43, 9.03) | <0.001 |
| HDL-C (mg/dL) | –4.98 (–6.19, –3.77) | <0.001 | –4.10 (–5.60, –2.60) | <0.001 |
| Triglycerides (log, fasting) | 0.284 (0.181, 0.388) | <0.001 | 0.309 (0.162, 0.455) | <0.001 |
| **Apolipoproteins** |  |  |  |  |
| ApoA (mg/dL) | –3.32 (–5.43, –1.22) | 0.002 | –2.76 (–5.43, –0.09) | 0.043 |
| ApoB (mg/dL) | 4.51 (2.73, 6.29) | <0.001 | 4.61 (2.45, 6.77) | <0.001 |
| **Inflammation** |  |  |  |  |
| CRP (mg/dL) | 0.124 (0.079, 0.169) | <0.001 | 0.124 (0.066, 0.182) | <0.001 |
| **Kidney Function Markers** |  |  |  |  |
| Serum Creatinine (mg/dL) | 0.121 (0.099, 0.143) | <0.001 | 0.100 (0.073, 0.127) | <0.001 |
| Urine Microalbumin (mg/dL) | –0.07 (–0.58, 0.45) | 0.802 | 0.09 (–0.59, 0.78) | 0.788 |
| Model 1: Unadjusted (univariate: OSA as single predictor). Model 2: Adjusted for marital status, education, employment, smoking status, and sleep duration. HbA1c models were additionally adjusted for history of diabetes, and lipid variables were adjusted for use of lipid-lowering medication.  Triglyceride was log-transformed, and regression analysis done for fasting participants (n =776).  Abbreviations: HbA1c= glycated hemoglobin A1C; LDL-C = Low-Density Lipoprotein Cholesterol; HDL-C = High-Density Lipoprotein Cholesterol; CRP = C-Reactive Protein. | | | | |

**Supplementary Figure S1. Distribution of dyslipidaemia by OSA status.** This figure displays the number and proportion of participants with and without dyslipidemia stratified by OSA risk category (0 = no/low OSA risk; 1 = intermediate-to-high OSA risk). Bars represent total counts, and overlaid labels show both raw counts and percentages within each dyslipidemia category. The figure provides an additional visual representation supporting the main findings that dyslipidemia was more common among individuals at intermediate-to-high OSA risk.
